# Domestic Economy in the Isolation Hospital

**Published:** 1916-02-26

**Authors:** 


### THE EDITOR'S LETTER-BOX.

### Some Interesting Problems.

**Our Correspondents are reminded that brevity of style and conciseness of statements greatly facilitate early insertion. We cannot in any way be responsible for the opinions expressed by correspondents, who must give their name and address as a guarantee of good faith, but not necessarily for publication.**

### Domestic Economy in the Isolation Hospital.

*To the Editor of* The Hospital.

Sir,—I have read with much interest the "Example of Prudent Housekeeping" in The Hospital of February 12, but there are several points I do not quite understand. One of these is how the extra percentage is reckoned out to cover the increased price of food. I am afraid I am very ignorant, but I confess that I can never understand what 15 per cent. or so means in plain figures, nor do I know how this estimate of additional cost is arrived at, though I seldom read a speech or an article on economy that does not reckon it out in this fashion. Another difficulty I found was in the allowance made for extra nurses. In my own small isolation hospital we constantly have to engage extra help, as we are very much understaffed, and if we have more than a dozen fresh admissions at the same time we cannot cope with them. I should very much like to know how to show my committee the exact extra cost entailed by these temporary nurses, and by the charwoman who must be engaged to wait upon them. I can quite bear out the statement that "the boarding of extra nurses entails more expense than that of the ordinary nurses," but you might have added that it increases the cost all round. I cannot account for this except that they seem to come in like visitors and have to be studied, or they would leave one in the lurch.

Lastly, I see that the cost of the patients' diet is reckoned out as if the amount on the diet sheet was all that has to be paid for. I have seen this calculation before, and I should like to know whether other matrons find the same that I do: that a good deal more is used than the exact quantities set down for the patients to get. For instance, if you have fifty typhoids on two quarts of milk a day each, no housekeeper would dare to order the exact 25 gallons of milk for them. There is some used in pouring from one can to another, some wasted in pouring from the can sent into each ward, and every sister knows how impossible it is to be left without any to fall back upon if a cup is spilt by accident. The same is true of bread and of other things. That is where I think paper calculations go wrong, but perhaps it is I who am in the wrong and other people may manage better. I should like to know.—Yours, etc.,

Another Isolation Matron.

